# Prevalence and Therapeutic Management of Infections by Multi-Drug-Resistant Organisms (MDROs) in Patients with Liver Cirrhosis: A Narrative Review

**DOI:** 10.3390/antibiotics11020232

**Published:** 2022-02-11

**Authors:** Lorenzo Onorato, Caterina Monari, Salvatore Capuano, Pierantonio Grimaldi, Nicola Coppola

**Affiliations:** Department of Mental Health and Public Medicine, Section of Infectious Diseases, University of Campania Luigi Vanvitelli, 80138 Naples, Italy; lorenzoonorato@libero.it (L.O.); caterina.monari@gmail.com (C.M.); salvatore.capuano2@studenti.unicampania.it (S.C.); pierantonio.grimaldi@studenti.unicampania.it (P.G.)

**Keywords:** liver cirrhosis, MDROs, bacterial infections, CRE, ESBL, MRSA

## Abstract

Bacterial infections are common events that significantly impact the clinical course of patients with cirrhosis. As in the general population, infections caused by multi-drug-resistant organisms (MDROs) are progressively increasing in cirrhotic patients, accounting for up to 30–35% of all infections. Nosocomial acquisition and prior exposure to antimicrobial treatment or invasive procedures are well-known risk factors for MDRO infections. Several studies have demonstrated that infections due to MDROs have a poorer prognosis and higher rates of treatment failure, septic shock, and hospital mortality. Due to the increasing rate of antimicrobial resistance, the approach to empirical treatment in cirrhotic patients with life-threatening infections has become significantly more challenging. In order to ensure a prompt administration of effective antibiotic therapy while avoiding unnecessary antibiotic exposure at the same time, it is of utmost importance to choose the correct antimicrobial therapy and administration schedule based on individual clinical characteristics and risk factors and rapidly adopt de-escalation strategies as soon as microbiological data are available. In the present paper, we aimed to provide an overview of the most frequent infections diagnosed in cirrhotic patients, the prevalence and impact of antimicrobial resistance, and potential therapeutic options in this population.

## 1. Introduction

Bacterial infections frequently complicate the clinical course of patients with liver cirrhosis [1,2]. It is estimated that up to one-third of cirrhotic subjects undergoing non-elective hospitalization receive a diagnosis of community-acquired or nosocomial infection [3], and the incidence is even higher among patients developing acute-on-chronic liver failure [4]. The occurrence of infections negatively impacts the natural history of patients with end-stage liver disease, significantly increasing the rate of hepatic decompensation and death [5].

During recent decades, the prevalence of infections due to multi-drug-resistant organisms (MDROs) among cirrhotic patients has increased over time, as demonstrated by a European multicenter cohort study [6], similarly to what has occurred in the general population. The spread of antimicrobial resistance poses a serious threat for public health; several studies have demonstrated that patients with infections due to MDR pathogens present a lower rate of effective empirical antimicrobial treatment, a longer length of hospital stay, and a higher mortality rate, compared with those infected with susceptible organisms [7,8]. Therefore, it is essential to choose the correct antimicrobial agents, taking into account the individual risk factors for MDR pathogens, and the correct administration schedules, combined with the adoption of early de-escalation strategies, as soon as microbiological data are available.

In the present paper, our aim was to provide an overview of the most frequent infections diagnosed in cirrhotic patients, the prevalence and impact of antimicrobial resistance, and potential therapeutic options in this population.

## 2. Bacterial Infections in Cirrhotic Patients

Bacterial infections are common clinical events that significantly impact the clinical course of patients with cirrhosis of all etiologies, involving up to 30% of hospitalized patients [2]. For example, Singal et al. [9] reported a prevalence of 23% in concomitant infections in 742,391 hospital admissions of patients with liver cirrhosis occurring in the United States from 1998 to 2007. Regarding the risk factors, a large multicenter prospective study conducted on 2864 cirrhotic patients undergoing nonelective hospitalization demonstrated that higher Model of end-stage liver disease (MELD) score, diabetes, and lactulose at admission were independent predictors of nosocomial infections [3]. Alcohol intake, multiple antibiotic courses, repeated hospital admissions, and invasive procedures were additional known risk factors [2].

Recently, a prospective cohort study enrolling 201 cirrhotic patients followed for a median of 36 months demonstrated that those developing bacterial infections presented a significantly higher risk of hepatic decompensation and death [5]. Moreover, Piano et al. in a prospective multicenter study collecting data from 1302 hospitalized cirrhotic patients with bacterial or fungal infections showed that the isolation of an MDR or XDR (extensively drug-resistant) pathogen was associated with an even higher 28-day mortality rate, compared with the isolation of drug-susceptible organisms (29% vs. 20%; *p* < 0.014) [7].

The most common infections involving cirrhotic patients include spontaneous bacterial peritonitis (SBP) (23–27% of cases), urinary tract infections (UTIs) (22–29%), pneumonia (19–20%), and skin and soft tissue infections (SSTIs) (8–12%); invasive fungal infections, mainly due to Candida spp, were reported in 5–10% of cases [2].

The clinical presentation of a bacterial infection in this setting can be misleading; for example, the fever may be absent because of the impairment of the immune response, especially in patients with advanced liver disease. Therefore, the clinician should maintain a high level of suspicion for an infection every time a cirrhotic patient presents with acute kidney injury, altered mental status, ascitic decompensation, or signs of acute-on-chronic liver failure. In the following sections, we will provide a brief description of their clinical characteristics.

### 2.1. Spontaneous Bacterial Peritonitis

SBP is defined by an ascitic neutrophil count of ≥250 cells/mm^3^ with a positive ascitic fluid bacterial culture without the evidence of an intra-abdominal, surgically treatable source of infection [10]. Bacterial cultures on ascitic fluid yield low sensitivity, so the presence of a neutrophil count ≥250 cells/mm^3^ with a negative culture result, usually defined as neutrocytic ascites, is a common finding, showing a mortality rate similar to culture-positive SBP and thus requiring equally prompt management. Symptoms include abdominal pain, vomiting, and diarrhea. Patients may also have signs of peritonitis or signs of systemic inflammation: fever, leukocytosis, tachycardia, or shock.

SBP may be asymptomatic in about a third of the patients. Therefore, diagnostic paracentesis should be performed in all patients with cirrhosis and ascites without delay at hospital admission and/or in patients with gastrointestinal bleeding, shock, signs of systemic inflammation, a worsening of liver or renal function, and hepatic encephalopathy [11]. Despite the suboptimal sensitivity of microbiological tests for the diagnosis of SBP, ascitic and blood culture should still be performed before starting antibiotics, as organisms can be isolated in 40–60% of cases [12]. Historically, Gram-negative bacteria, especially *E. coli*, *Proteus,* and *K. pneumoniae*, have been considered the leading cause of SBP; however, in recent years, a progressive increase in the prevalence of Gram-positive strains, such as *Enterococcus* spp. and *Staphylococcus aureus*, have been observed [10].

### 2.2. Urinary Tract Infections

As stated before, UTIs are the second most common infections in cirrhotic patients. In fact, residual urinary volume and vesical dysfunction are common in this population. Moreover, recurrent hospitalization and catheterization of cirrhotic patients are associated with nosocomially acquired UTIs [13]. In the study by Fernandez et al. [6], it was found that urinary catheterization during hospital admission was associated with MDR bacterial infections, making the withdrawal of the catheter one of the priorities as soon as the clinical conditions allow.

### 2.3. Pneumonia

Among the diagnosed infections in hospitalized cirrhotic patients, pneumonia is the one with the highest risk of mortality [14]. Compared with noncirrhotic patients, in cirrhotic patients, community-acquired pneumonia (CAP) more frequently presents multilobar involvement, impaired consciousness, renal failure, and septic shock [15].

Lower respiratory tract infections (LRTIs) are common complications occurring in patients with liver cirrhosis during hospitalization. In the study conducted by Bajaj et al. [3], the prevalence of LRTIs among cirrhotic patients with nosocomial infections was 17%, compared with 9% among subjects with community-acquired infections. The main risk factors for hospital-acquired pneumonia (HAP) in this setting are represented by hepatic encephalopathy, mechanical ventilation, previous antimicrobial, and use of proton pump inhibitors [13].

The microbiology is overall similar to that of the general population, with *Streptococcus pneumoniae* being the first agent involved in CAP, while Staphylococci and Gram-negative bacilli are the leading cause of HAP [14]. In a study by Piano et al. [7], respiratory tract infections were independently associated with an etiology due to MDR (OR 3.20 CI 1.83–5.59, *p* < 0.001) or XDR pathogens (OR 2.71, CI 1.29–5.70, *p* = 0.009).

### 2.4. Skin and Soft Tissue Infections (SSTIs)

Necrotizing fasciitis is the most alarming SSTI, potentially presenting with septic shock and bacteremia ab initio, requiring early surgical intervention in order to decrease morbidity and mortality [16]. The most common causative isolates are Gram-positive bacteria (group A *Streptococci* and *Staphylococcus aureus*), but Gram-negative organisms (including *E. coli*, *Klebsiella* spp., *P. aeruginosa*, *Aeromonas* spp., *Vibrio* spp.) are also frequently reported in patients with cirrhosis [17].

## 3. Multi-Drug-Resistant Organisms (MDROs): Mechanisms of Resistance and Prevalence in Cirrhotic Patients

Infections caused by MDROs are progressively increasing in patients with liver cirrhosis [12,18,19]. Fernandez et al. described a rising prevalence over time of infections due to MDROs among patients with cirrhosis in Spain, with a rate of infections caused by MDR bacteria increasing from 18% in 2005–2007 to 23% in 2010–2011 [20]. In 2018, Bartoletti et al. reported that MDROs accounted for almost one-third of bloodstream infections (BSIs) (31%) in patients with liver cirrhosis [8]. Additionally, Fernandez et al. described a similar rate (23.3–28%) in a retrospective evaluation of two series of patients with decompensated cirrhosis in Europe, with a significantly higher total number of MDR infections in northern and western Europe (19.4% and 19.3%, respectively vs. 9.7%, *p* < 0.001) and high heterogeneity in the different countries. Another recent prospective multicenter cohort study by Piano et al. found a global 34% prevalence of infections due to MDR pathogens [7]. A different geographical distribution of MDR bacterial infections has been described, with a peak rate of 50% in Asia, especially in India (73% of isolates), compared with lower frequencies in North America (16% in the US and 24% in Canada).

The site of acquisition of infections determines the risk of MDROs, with higher rates of infections acquired in the healthcare environment: 36% in nosocomial, 32% in healthcare-associated (HCA) infections, and 4% in community-acquired (CA) infections (*p* < 0.001) [6]. Moreover, compared with infections caused by susceptible microorganisms, those caused by MDROs have a poorer prognosis and higher rates of treatment failure, septic shock (26% vs. 10%, respectively), and hospital mortality (25% vs. 12%, respectively) [12]. MDROs were more commonly isolated in intensive care units (ICUs) (23.8% vs. 12.2%; *p* = 0.005) and caused more frequently severe sepsis/shock (30.3% vs. 12.2%, *p* < 0.001) or acute-on-chronic liver failure (20.5% vs. 9.4%, *p* < 0.001) in a subsequent study conducted by the same authors [6]. The poor outcome related to infections due to MDROs among cirrhotic patients was confirmed in other studies [7,19].

Aside from the poorer outcome, the importance of the increased prevalence of MDRO infections lies in the choice of antibiotic empirical therapy and the consequences of its failure [6]. Antibiotic resistance was associated with the failure of antibiotic strategies since they were based mainly on third-generation cephalosporins (3GCs) or quinolones.

In this section, we describe the most frequent MDROs responsible for bacterial infections in this vulnerable population, their prevalence, and the main mechanisms of antibiotic resistance. The characteristics of the most recent studies evaluating the prevalence of MDROs in cirrhotic patients are listed in Table 1.

### 3.1. Gram-Positive Bacteria Infections in Cirrhotic Patients

Recently, a change in the bacterial ecology has interested the field of infections in cirrhotic patients. Gram-positive cocci and healthcare-associated infections now play increasingly important roles, partly replacing community-acquired infections and infections due to Gram-negative bacteria, which, in the past, represented together almost 70% of infections. Gram-positive cocci now account for up to 40% of all bacterial infections in cirrhotic patients. Two reasons were mainly behind this shift: First, there has been increasing use of procedures in the care of these patients that can increase the exposure to this kind of infection, such as endoscopic band ligation, portosystemic intrahepatic transjugular shunt, percutaneous treatment, and chemoembolization for hepatocellular carcinoma, transjugular hepatic biopsy, etc.; furthermore, there was an increasing possibility that advanced stage cirrhotic patients were admitted to intensive care units (ICUs) and were, therefore, exposed to the infective risks of these departments and the related procedures [22].

In a worldwide study by Piano et al., the prevalence of Gram-negative isolates was 57%, 38% were Gram-positive bacteria, and 4% fungi. In Europe and America, more Gram-positive bacteria were isolated, compared with Asia (43% and 37% vs. 27%, respectively) [7].

As regards MDR and XDR Gram-positive, the most common are methicillin-resistant *Staphylococcus aureus* (MRSA), and vancomycin-resistant Enterococci (VRE) [22]. In the study by Piano et al. [7], MDR bacteria are shown to be more often a cause of infection in cirrhotic patients in Asia (51%) than in Europe or America (29% and 27%, respectively).

In general, the worldwide context reveals Gram-negative MDR and XDR species as predominant, apart from Korea, where MRSA appears to be the most frequent [22].

#### 3.1.1. *Staphylococcus aureus*

*S. aureus* belongs to the Genus Staphylococcus of the family Staphylococcaceae. It is a major human pathogen, characterized by high infection and mortality rates, able to acquire resistance to any antibiotic. As stated, MRSA is among the most important MDR bacteria, estimated to be responsible for over 148,000 infections and 7000 deaths in the EU in 2015 [23].

Originally well susceptible to penicillin, it quickly developed resistance a few years after the introduction of the drug. The target of β-lactam antibiotics, such as penicillin, is the transpeptidase moiety of the penicillin-binding protein-2 (PBP) [24]. *S. aureus* obtains penicillin resistance by producing a plasmid-encoded penicillinase able to hydrolyze a β-lactam ring necessary for the drug’s antimicrobial activity. Methicillin is semisynthetic penicillin synthesized in 1959 to overcome the first penicillin-resistance wave. Shortly after its introduction in 1961, the first methicillin-resistant *S. aureus* appeared in the UK, showing also resistance to cephalosporins and carbapenems; this ability was acquired owing to the production of an additional PBP, called PBP2a, which has a reduced affinity to β-lactam antibiotics [25]. The serine targeted by β-lactams is hidden in a deep pocket, not accessible by any antibiotic of the family besides ceftaroline and ceftobiprole, fifth-generation cephalosporins designed for this specific purpose, able to induce a conformational change that uncovers the targeted serine [24].

Vancomycin is a glycopeptide antibiotic, the gold standard for MRSA. However, the diffusion of MRSA led to the use of vancomycin on an increasingly large scale, which brought about the selection of vancomycin-intermediate (VISA) and vancomycin-resistant *S. aureus* (VRSA), the former with a MIC of 4–8 μg/mL, the latter characterized by a MIC > 16 μg/mL. The vancomycin target is the d-Ala–d-Ala residues of the peptidoglycan precursors. VISA, first observed in Japan in 1996, presents a thickened cell wall rich in non-cross-linked peptidoglycan chains, offering d-Ala–d-Ala residues on its external surface so that vancomycin is blocked on the cell wall and cannot reach its true target, which is located within the cell. VRSA, instead, acquired its mechanism of resistance from vancomycin-resistant enterococci. The six different strains of VRSA identified in the USA all present the vanA gene, which encodes for a modified peptidoglycan precursor presenting a terminal d-Ala–d-Lac, with little vancomycin affinity. This terminus confers resistance to all glycopeptides [25].

*S. aureus* presents resistance to a long series of other antibiotics. The introduction of ciprofloxacin shortly led to the selection, especially in MRSA, of species mutated in subunit gyrB of DNA gyrase and grIA of topoisomerase IV, or the overexpression of the efflux pump NorA, all mechanisms of fluoroquinolone resistance. Linezolid and daptomycin are two widely used anti-MRSA agents, the latter especially for lung infections and the former for skin and soft tissue infections and endocarditis. Linezolid, of the oxazolidinone family, interferes with protein synthesis by binding to domain V of the 23S subunit of the bacterial ribosome. The resistance to this drug is due to a mutation in its binding site. Daptomycin binds the bacterial cytoplasmic membrane in the presence of calcium ions. Resistance mechanisms to this drug include variations in the membrane voltage difference and alterations of its binding sites. Sulfonamide inhibits dihydropteroate synthase (DHPS), and trimethoprim target is dihydrofolate reductase (DHFR); they are clinically used only in combination. Mutation in DHPS prevents the drug from binding to the enzyme, and acquisition of the dfrA gene leads to the production of a DHFR enzyme not susceptible to inhibitions, producing resistance to trimethoprim–sulfamethoxazole, an otherwise useful alternative anti-staphylococcal agent. Tetracyclines are a family of protein synthesis inhibitors alternatively used against *S. aureus* species. Resistance to this 30s subunit-binding drug occurs due to an acquisition of efflux pumps (plasmidic genes *tet*(K) and *tet*(L)) and ribosome protection (genes *tet*O/M) [25].

Considering the study conducted by Fernandez et al. [6], in cirrhotic patients, *S. aureus* accounted for 10.5% of all isolated pathogens, with a 40% MRSA prevalence. Piano et al. [7] reported an overall prevalence of methicillin resistance of 24% in 54 clinical isolates of *S. aureus*. Similarly, in the study published by Bartoletti et al. [8], 12 out of 53 (22.6%) *S. aureus* strains isolated from blood cultures of cirrhotic patients were resistant to oxacillin.

#### 3.1.2. *Enterococcus* spp.

*Enterococcus faecium* and *Enterococcus faecalis* are pathogens characterized by intrinsic resistance to various antibiotics, including β-lactams. Nonetheless, they are able to acquire a series of resistance mechanisms and even to transmit their abilities to different species, as described above for VRSA [24]. VRE, the most relevant isolates of *E. faecium* and *E. faecalis*, caused more than 16,000 infections and 1000 deaths in the European Union in 2015 [23].

Enterococci PBP shows a low affinity for β-lactam antibiotics. Among them, enterococci have a different degree of sensitivities to different classes—it is most sensitive to penicillin and ampicillin (especially *E. fecalis*, even though at higher MICs than the other cocci), less to carbapenems, and completely resistant to cephalosporins. The best example of the low affinity of enterococci PBPs to β-lactams is the PBP5, which confers complete resistance to cephalosporins. Enterococci can acquire PBP mutations able to confer high-level resistance to penicillins, such as PBP5 in *E. faecium*. Enterococci are intrinsically resistant to aminoglycosides due to the impossibility of this drug penetrating their cell wall. This is not true when they are used with a cell wall synthesis inhibitor such as ampicillin. *E. faecium* is also able to enzymatically inhibit aminoglycosides through acetyltransferases and phosphotransferases. Drugs inhibited in this way include tobramycin, kanamycin, and amikacin. Additionally, gentamycin resistance can be acquired through enzymes that phosphorylate and acetylate the antibiotic, making it unfit to bind to the 30S subunit.

Due to their ability to absorb folate from the environment, Enterococci are naturally resistant to trimethoprim–sulfamethoxazole (although in vitro they can appear sensitive). *E. faecalis* can be resistant to clindamycin using an efflux pump, while *E. faecium* can acquire the *lin*B gene that encodes for an enzyme able to adenylate and inactivate the drug. Tetracycline resistance mechanisms include the production of efflux pumps and ribosomal protection proteins [24].

Intuitively, VRE has an identical mechanism of resistance to VRSA, since the latter is acquired from the former. In addition to VanA, also found in VRSA, the operon VanB has been documented. VanB, differently from VanA, does not confer teicoplanin resistance. As far as the resistance to daptomycin is concerned, the different species have a different mechanism: *E. faecium*, similarly to *S. aureus*, increases the positive charge of the membrane using electrostatic repulsion of the daptomycin–calcium complex. *E. faecalis* uses a different mechanism, based on the concept that the daptomycin–calcium complex mainly binds the cell membrane at the division septum plane and that cardiolipin enables the drug to reach the inner layer. When there are mutations in the LiaSFR signaling system, cardiolipin is positioned in nonseptal locations of the membrane, and therefore, daptomycin cannot oligomerize in the septal area. Linezolid is a drug of choice in VRE. The enterococci can become resistant to it in a similar way to *S. aureus*, by developing mutations in the 23 s subunit [24].

Among the cohort evaluated by Fernandez et al. [6], *E. faecalis* accounted for 10% and *E. faecium* for 5% of all microbiologically documented infections. In particular, 2 out of 14 (14.3%) *E. faecium* isolates showed resistance to vancomycin. In the study by Piano et al. [7], 91 enterococci were isolated, with an overall prevalence of ampicillin and vancomycin resistance of 42% and 18%, respectively.

### 3.2. Gram-Negative Bacteria Infections in Cirrhotic Patients

#### Enterobacteriaceae

Enterobacteriaceae is a large family of Gram-negative bacteria that belongs to the order Enterobacterales, including more than 120 species of bacteria. The most common Enterobacteriaceae are *Escherichia* spp., *Klebsiella* spp., *Citrobacter* spp., and *Enterobacter* spp.

Among patients with cirrhosis, *Escherichia coli* and *Klebsiella pneumoniae* are responsible for up to 50% of all bacterial infections. They are the most frequently isolated pathogens in spontaneous bacterial peritonitis, UTIs, and primary BSIs [26].

The main mechanisms of antibiotic resistance of these bacteria are represented by enzymatic, i.e., production of β-lactamases, or by nonenzymatic mechanisms, such as porin deficit and drug efflux pump overexpression, or by a combination of them. Both mechanisms may intrinsically be expressed (e.g., chromosomal genes) or acquired.

The production of β-lactamases that hydrolyze the β-lactam ring is the predominant mechanism of resistance to β-lactams among Enterobacteriaceae [27,28]. β-lactamases are classified according to molecular (Ambler Classification) [29] or functional (Bush, Jacoby, and Medeiros Classification) [30] characteristics. Ambler classification is based on amino acid sequences and divides β-lactamases into four molecular classes: class A (extended-spectrum β-lactamases (ESBL), carbapenemase (KPC)), B (metallo-β-lactamases (MBLs)), C (AmpC cephalosporinases), and D (oxacillinases). Class A, C, and D proteins are serine enzymes, whereas those of class B are metalloenzymes, containing one or two zinc ions. The functional classification by Bush, Jacoby, and Medeiros allows correlating the different enzymes to their clinical role, i.e., providing selective resistance to specific classes of β-lactams. This classification divides β-lactamases into four functional groups (1-4) and subgroups, defined by letters, according to different substrate hydrolysis and inhibitor profiles (clavulanic acid, sulbactam, tazobactam, avibactam) [31]. However, the combination of both criteria enabled a more comprehensive classification of the 4 major β-lactamases, encompassing 17 functional groups.

Ambler class A β-lactamases comprise the largest number of enzymes, with a very wide spectrum of activity.

ESBLs belong to Ambler class A and to Bush–Jacoby–Medeiros functional group 2be; most representative types are SHV, TEM, and CTX m [32]. ESBL confers resistance to penicillin, third- and fourth-generation cephalosporins (3GCs and 4GCs, respectively), and monobactams [33]. ESBL-producing Enterobacteriaceae are frequently resistant also to non-β-lactams such as fluoroquinolones, trimethoprim–sulfamethoxazole, and aminoglycosides [34]. Most ESBLs are susceptible to “old” and “new” β-lactam inhibitors (BLI) that have been approved by the FDA. ESBL-producing Enterobacteriaceae, in particular *Escherichia coli*, are the main MDR pathogens identified in patients with liver cirrhosis [6,7].

Fernandez et al. described an increasing rate over decades of infections caused by ESBL-producing Enterobacteriaceae from 1.2% in 2002 to 7.5–8.7% in 2005-2011 [20]. More recently, the prospective multicenter cohort study by Bartoletti et al. reported a prevalence of BSI caused by ESBL-producing Enterobacteriaceae of 14% [8]. However, in 2019, Piano et al. [7] described a much higher overall frequency of infections due to 3GC-resistant Enterobacteriaceae, with a rate of 35%.

Class A serine-carbapenemases (functional group 2f, according to Bush–Jacoby–Medeiros classification) include several enzymes, among which the most relevant are *Klebsiella pneumoniae* carbapenemase (KPC), Serratia marcescens enzyme (SME), IMIpenemase (IMI), and certain variants of GES enzymes [27,35]. They may be chromosomally encoded (SME), plasmid encoded (KPC and GES), or both (IMI). KPC has been mainly detected in *Klebsiella pneumoniae* but also in other species of Enterobacteriaceae. All class A carbapenemases confer resistance to carbapenems; they are not inhibited by clavulanic acid or tazobactam but are inhibited by new β-lactamase inhibitors, such as avibactam and vaborbactam [36].

MBLs (Ambler class B or Bush–Jacoby–Medeiros functional group 3) are characterized by the presence of two zinc ions at the active site. They encompass the B1 subclass enzymes New Delhi MBL (NDM), Verona IMipenemase (VIM), and IMipenem-resistant Pseudomonas (IMP), and may be plasmid encoded or acquired [27]. MBLs hydrolyze most β-lactams, including carbapenems, but unlike class A carbapenemases, they do not hydrolyze monobactams and are not inhibited by β-lactamase inhibitors [32], including avibactam and vaborbactam.

Oxacillinases (Ambler Class D or Bush–Jacoby–Medeiros functional group 2d) have greater hydrolytic activity against oxacillin, and some of them can hydrolyze cephalosporins and carbapenems. OXA enzymes are weakly inhibited by clavulanic acid, but some are inhibited by tazobactam and avibactam [32].

As regards carbapenemases, the study by Bartoletti et al. [8] reported a prevalence of BSI caused by carbapenemase-producing Enterobacteriaceae of 3% in a cohort of patients with liver cirrhosis. Piano et al. [7] reported a global prevalence of Enterobacteriaceae resistance to carbapenems (CRE) among patients with cirrhosis of 9%, with a disproportionately higher rate in Asia (20%), especially in India (36%). Moreover, a mild increase in the rate of carbapenem-resistant Enterobacteriaceae was observed by Fernandez et al. between 2011 and 2017–2018 [6]. Regarding specific pathogens, the same study described an 0.4% prevalence of infections due to carbapenem-resistant *Klebsiella pneumonia*; the higher rate was reported in the UK (6%). Carbapenem-resistant Enterobacteriaceae were the pathogens with the highest mortality rate (44%) among patients with BSI and liver cirrhosis.

AmpC cephalosporinases belong to Ambler class C (Bush–Jacoby–Medeiros functional group 1). They confer resistance to most cephalosporins, including 3GC such as cefotaxime and ceftriaxone, as well as to cephamycins, such as cefoxitin [32]. They are also able to hydrolyze penicillins and some monobactams. There are chromosomal- or plasmid-mediated AmpC cephalosporinases. AmpCs are usually encoded by chromosomal AmpC genes in *Citrobacter* spp., *Enterobacter* spp. and *Serratia* spp., whereas plasmid-borne enzymes are more prevalent among *Klebsiella* and *Salmonella* spp. The production of AmpC enzymes may be low (“repressed”) or inducible (“derepressed”), in particular after exposure to β-lactams [27].

Studies reporting data on the prevalence of infections due to Enterobacteriaceae-producing AmpC enzymes are still few. The prospective study by Fernandez et al., mentioned above, described an overall rate of infections due to AmpC-producing *Enterobacter* spp. of 1.2%, with a peak rate of 6% in France [6].

### 3.3. Nonfermenting Gram-Negative Bacteria

Nonfermenting Gram-negative bacteria (NFGNB), such as *Pseudomonas aeruginosa*, *Acinetobacter baumannii*, and *Stenotrophomonas maltophilia*, are bacteria that can cause important nosocomial infections with significant morbidity and mortality. In this section, we discuss the main mechanisms of resistance of *Pseudomonas aeruginosa* and *Acinetobacter baumannii* and their prevalence in cirrhotic patients.

*P. aeruginosa* has a wide variety of mechanisms of antibiotic resistance, including chromosomal determinants and intrinsic and adaptive resistance. The main mechanisms of resistance are the overexpression of constitutive or inducible drug efflux pumps, modification of membrane permeability, and the production of β-lactamases (inducible AmpC cephalosporinase) [37].

Porin loss is a significant mechanism of resistance of *P. aeruginosa*, with the loss of outer membrane protein D (OprD) being one of the major mechanisms of carbapenem resistance. Often the modification of outer membrane permeability is associated with the expression of drug efflux pumps; the four main efflux pumps found in *P. aeruginosa* are MexAB-OprM, MexCD-OprJ, MexEF-OprN, and MexXY [38,39]. Regarding intrinsic mechanisms of resistance of *Pseudomonas aeruginosa*, β-lactamase production plays a key role in the natural resistance of this bacteria to aminopenicillins and some cephalosporins; inducible AmpC expression confers reduced susceptibility to imipenem, and the constitutive expression of the MexAB-OprM and MexXY efflux pumps confers lower levels of susceptibility to the majority of β-lactams, except for imipenem, and fluoroquinolones, and to aminoglycosides, respectively [37,40]. Despite the so-called intrinsic resistome, *P. aeruginosa* is characterized by the ability to develop antimicrobial resistance through the acquisition of chromosomal mutations, leading to an overproduction of chromosomal AmpC cephalosporinases, to a structural modification of AmpC, or to up-/downregulation of specific efflux systems and porins. These mechanisms, or a combination of them, are responsible for the *P. aeruginosa* profiles resistant to the main classes of antibiotics [41]. For example, resistance to antipseudomonal β-lactams is mainly mediated by the association of OprD inactivation and AmpC overexpression [42], whereas fluoroquinolone resistance with mutations in DNA gyrases (GyrA and GyrB) and type IV topoisomerases [43], and colistin resistance with the modification of the lipid A moiety of lipopolysaccharide [44]. Lastly, another peculiar characteristic of *P. aeruginosa* of increasing concern is the acquisition of transferable β-lactamases, such as ESBL and carbapenemase, especially MBLs, with VIM and IMP types being the most frequently reported [37].

*A. baumannii* is a Gram-negative bacterium that causes nosocomial infections with high mortality rates and represents a global threat and a therapeutic challenge due to increasing antimicrobial resistance [45]. *Acinetobacter baumannii* antibiotic resistance can be mediated by three main mechanisms, i.e., control of antibiotic transportation through membranes (reduction in porin permeability or increased efflux pumps), modification of antibiotic targets, and enzymatic inactivation. *A. baumannii* is intrinsically resistant to penicillins and cephalosporins [46]. Resistance to β-lactam antibiotics can be conferred through all the abovementioned mechanisms, but the most significant mechanism of resistance of *A. baumannii* is by the production of OXA-type β-lactamases, which hydrolyze carbapenems. The most prevalent OXA enzymes in *A. baumannii* strains are OXA-23, OXA-24, OXA-40, OXA-58, OXA-143, and OXA-235. A. baumannii can also produce Ambler class B β-lactamases, such as IMP, VIM, and NDM [47].

Few studies report the prevalence of infections due to NFGNB among cirrhotic patients. NFGNB (*P. aeruginosa*, *A. baumannii*, *S. maltophilia*) were isolated in 15% of BSI episodes in the cohort evaluated by Bartoletti et al. in 2014 [21]. The same authors, in a more recent prospective study [8], described a prevalence of BSI due to *P. aeruginosa*, *A. baumannii*, and *S. maltophilia*, of 5%, 1%, and 1%, respectively. Nevertheless, susceptibility profiles of these bacteria were not available. Lastly, the multicenter study conducted by Gernandez et al. in Europe described an 0.8% prevalence of infections caused by both carbapenem-resistant *P. aeruginosa* and *A. baumannii* [6].

## 4. Risk Factors Associated with MDRO Infections

Considering the poor outcome related to infections by MDROs in patients with liver cirrhosis, several studies have investigated what factors are associated with the development of infections due to MDROs [6,7,8].

Among known risk factors for bacterial infections in cirrhotic patients, poor liver function, variceal bleeding, low ascitic protein levels, prior SBP, and recent hospitalization are the most reported in the literature [18]. Among specific factors associated with infections caused by MDROs, a multinational cohort study conducted by Piano et al. identified the use of systemic antibiotics for at least 5 days in the previous 3 months (OR 1.92, 95% CI 1.32–2.80, *p* = 0.001), exposure to healthcare (healthcare-associated infections OR 1.62, 95% CI 1.04–2.52, *p* = 0.032; nosocomial infections OR 2.65, 95% CI 1.75–4.01, *p* < 0.001), and site of infection, i.e., UTIs (OR 2.48, 95% CI 1.59–3.87, *p* < 0.001), pneumonia (OR 3.20, 95% CI 1.83–5.59, *p* < 0.001), and skin and soft tissue infections (OR 2.92, 95% CI 1.41–6.09, *p* = 0.004) [7].

Fernandez et al. confirmed nosocomial infections (OR 2.74, 95% CI 1.45–5.19, *p* = 0.002), ICU admission (OR 2.09, 95% CI 1.11–3.96, *p* = 0.02), and recent hospitalization (OR 1.93, 95% CI 1.04–3.58, *p* = 0.038) as factors related to MDROs [6]. Additionally, Bartoletti et al. [8], in the multivariate analysis, adjusted for clinical severity and length of in-hospital stay before the onset of BSI, found that prior (<30 days) antimicrobial therapy (OR 2.91; 95% CI 1.73–4.88; *p* < 0.001) and prior (<30 days) invasive procedures (OR 2.51; 95% CI 1.48–4.24; *p* = 0.001) were associated with a higher risk of infections caused by MDROs, whereas SB*P* as a source of BSIs was related to lower risk (OR 0.30; 95% CI 0.12-0.73; *p* = 0.008). Lastly, other factors reported to be independently associated with the development of multiresistant infections were recent infections by MDR bacteria in the last 6 months (HR hazard ratio 2.45; 95% CI:1.04–5.81), especially previous infection by ESBL-producing *E. coli*, and the use of β-lactams in the last 3 months (HR, 2.39; 95% CI: 1.18–4.85; *p* = 0.02) [20].

Interestingly, in the study conducted by Piano et al. [7], previous antibiotic prophylaxis for SB*P* with quinolones was not found to be more frequent in patients with MDRO infections, contrary to what emerged in a previous study by Fernandez et al. (HR, 2.69; 95% CI: 1.36–5.30; *p* = 0.004).

## 5. Principles of Antimicrobial Treatment

Given the increasing rate of antimicrobial resistance in the general population, the approach to empirical treatment in patients with life-threatening infections has become significantly more challenging.

On the one hand, it is well known that delayed administration of effective antibiotic therapy in critically ill patients is associated with an increase in in-hospital mortality [48], and this correlation has been clearly demonstrated also in cirrhotic subjects [6]. A prospective cohort study conducted in 19 European centers on 312 cirrhotic patients with BSIs reported a significantly higher 30-day mortality rate among those who did not receive adequate antimicrobial treatment within 24 h from the index blood culture [8]; furthermore, the authors reported a close correlation between the isolation of an MDRO and the probability of receiving inappropriate empirical treatment. Similar results have been found in a multicenter study published by Piano et al. in 2019 [7].

On the other hand, the consumption of broad-spectrum antibiotics is one of the most important drivers of antimicrobial resistance [49], and every effort should be made to avoid unnecessary prescriptions and limit the further selection of resistance.

To conciliate these conflicting needs, it is of utmost importance to choose the correct antimicrobial therapy based on individual clinical characteristics and risk factors for infections due to MDROs. In this view, it is important to underline the need to collect the microbiological specimens before starting the empiric antimicrobial therapy; in fact, the identification of the microorganism and its antimicrobial susceptibility will allow an antibiotic de-escalation and reduce the selective pressure on microorganisms.

## 6. Empirical Antimicrobial Schedules

In the following section, we discuss the main treatment options for the most common infections diagnosed in cirrhotic patients.

The possible schedules of empirical antimicrobial therapy for the most common infections in cirrhotic patients are listed in Table 2.

For SBP, a third-generation cephalosporin remains the recommended choice for community-acquired infections [50], although a small controlled randomized trial published in 2000 demonstrated the noninferiority of amoxicillin–clavulanic acid, compared with cefotaxime, in this setting. However, the empirical treatment, particularly in severe cases, should be tailored on the basis of local epidemiology, the presence of potential risk factors for MDRO infections, and previously known colonization. A meta-analysis published in 2018 reported a similar prevalence of resistance to third-generation cephalosporins among community-acquired and nosocomial SBP [51] in studies published over the previous 10 years.

Regarding healthcare-associated and nosocomial infections, piperacillin–tazobactam or a carbapenem, particularly in critically ill patients treated in settings with a high prevalence of ESBL-producing Enterobacteriaceae, should be considered in this setting. Moreover, the addition of an antimicrobial agent active against MDR Gram-positive bacteria, such as vancomycin, linezolid, or daptomycin, should be considered in areas with a high prevalence of methicillin-resistant *S. aureus* or ampicillin-resistant *Enterococcus* spp. [50]. For example, a randomized, controlled trial conducted by Piano et al. [52] demonstrated a higher efficacy of meropenem and daptomycin combination, compared with ceftazidime monotherapy in 31 patients with nosocomial SBP. However, we should consider that the narrow therapeutic index and nephrotoxicity of vancomycin may limit its use in cirrhotic patients, and doses may need an adjustment based on therapeutic drug monitoring. Moreover, in settings with a high prevalence of vancomycin-resistant *E. faecium*, daptomycin or linezolid should be preferred, with particular attention for the latter to the occurrence of thrombocytopenia.

For other infections, such as UTI, pneumonia, and SSTI infections, the recommendations may be similar to those provided by the international guidelines for the general population.

In particular, for uncomplicated community-acquired UTIs, fosfomycin and co-trimoxazole are valuable options, while an aminopenicillin combined with a β-lactamase inhibitor or a third-generation cephalosporin can be considered in septic patients. Regarding the nosocomial infections, fosfomycin and nitrofurantoin are still potentially useful for uncomplicated infections; in severe diseases, piperacillin–tazobactam or carbapenem should be prescribed.

In patients with community-acquired pneumonia, the combination of amoxicillin–clavulanic acid or ceftriaxone with a macrolide is the most commonly used approach, while for hospital-acquired or ventilator-associated pneumonia the readers should refer to the indications reported in the international guidelines [53,54].

Amoxicillin–clavulanic acid can be administered for non-necrotizing community-acquired SSTI, with the addition of cotrimoxazole or doxycycline, in the case of a high prevalence of community-acquired MRSA; in patients with necrotizing fasciitis, piperacillin–tazobactam, or meropenem for nosocomial infections in settings with a high prevalence of ESBL-producing bacteria, in combination with an anti-MRSA agent, is recommended by the Infectious Diseases Society of America (IDSA). When not using linezolid, the addition of clindamycin can be considered for the antitoxin activity [55].

## 7. Pharmacokinetic Considerations in Cirrhotic Patients

It is known that, in decompensated cirrhotic patients, the pharmacokinetics of several drugs, including antibiotics, can significantly change [56,57]. The alteration of hepatic enzymatic activity and the reduced liver blood flow, combined with the expansion of the extracellular fluid volume, the hypoalbuminemia, and hyperbilirubinemia frequently found in cirrhotic patients, can deeply alter the exposure to antimicrobial agents, particularly for the hydrophilic molecules and for those with high plasma protein binding.

If we add these pharmacokinetic alterations to those commonly found in critically ill patients [58], we can understand why the optimization of antimicrobial drug administration is of essential importance in this setting. In particular, β-lactams are strictly time-dependent antibiotics and exert higher antimicrobial activity when administered in continuous or extended infusion [59]. There is a growing body of evidence demonstrating the superiority of these administration strategies over intermittent infusion in critically ill patients [60]. A prospective multicenter cohort study confirmed these findings also in 119 patients with end-stage liver diseases treated for BSIs [61]. The study reported a lower 30-day mortality rate in patients receiving either piperacillin–tazobactam or meropenem in continuous or extended infusion, compared with those treated with the same drugs in intermittent dosing. These data highlight the importance of optimizing the infusion strategies of antimicrobials in this difficult-to-treat population. Particular attention should be given to the dosing of meropenem; a prospective cohort study conducted in 2020 among 54 Spanish patients with decompensated cirrhosis demonstrated that meropenem clearance is significantly reduced in patients with higher MELD score, as well as the presence of an acute on chronic liver failure can affect the volume of distribution of the drug [62]. A therapeutic drug monitoring (TDM) could be considered in this setting to ensure adequate exposure when treating infections due to MDROs. The use of TDM is clearly indicated for drug classes with narrow therapeutic spectra and a high potential for nephrotoxicities, such as glycopeptides or aminoglycosides, as recommended by international guidelines for the general population [63]. Impaired renal function is commonly found in cirrhotic patients, particularly during a bacterial infection, resulting in a higher risk of side effects due to drug accumulation. A retrospective study among 201 cirrhotic patients treated with vancomycin reported a supratherapeutic drug concentration in 33% of Child–Pugh A patients, and up to 50% in Child–Pugh B and C classes, even though most of them received lower dosing than that recommended on the basis of their actual body weight and creatinine clearance [64]. Furthermore, drugs for which dosing modification based on renal function is not routinely recommended could also be beneficial for TDM in this population. For example, a case–control study including 52 patients receiving linezolid treatment demonstrated that the occurrence of plasma overexposure or drug discontinuation due to hematological side effects were more frequent among cirrhotic, compared with noncirrhotic subjects [65]. Thus, careful monitoring is warranted, and alternative better-tolerated options should be considered when treating cirrhotic patients, especially those with acute kidney injury.

## 8. De-Escalation Policies and Duration of Treatments

If the choice of the correct empirical antibiotic treatment and dosing strategy is important to increase the survival of patients with life-threatening infections, it is equally important to re-evaluate the therapy as soon as the microbiological data are available, in order to avoid unnecessary prescriptions and limit the selection of resistance.

Although no specific data are available for cirrhotic patients, a meta-analysis including three randomized, controlled trials and 16 observation studies demonstrated that patients with pneumonia or BSI undergoing de-escalation show a similar, or even lower, mortality rate, compared with those continuing the empirical broad-spectrum therapy [66]. A recently published consensus statement of the European Society of Intensive Care Medicine (ESICM) and the European Society of Clinical Microbiology and Infectious Disease (ESCMID) recommends the adoption of de-escalation policies to all critically ill patients receiving antimicrobial treatment [67]. Thus, the available evidence demonstrates that de-escalation is a safe and effective approach that can also be applied for difficult-to-treat patients, such as cirrhotic subjects with severe infections.

## 9. Conclusions

Bacterial infections are potentially life-threatening events that commonly complicate the clinical course of patients with end-stage liver disease. The progressive increase in the rate of antimicrobials observed in the general population in recent decades has also involved cirrhotic patients, who are frequently hospitalized and are prescribed broad-spectrum antibiotic treatments. In order to start early effective treatment to critically ill patients while limiting a further selection of resistance at the same time, it is of utmost importance to apply an approach based on the evaluation of individual risk factors for MDR pathogens when choosing the correct agents and administration schedules for empirical treatment, as well as adopting a rapid de-escalation policy on the basis of the microbiological data.

## Figures and Tables

**Table 1:** Characteristics of studies evaluating the prevalence of MDR pathogens in cirrhotic patients.

First Name, Year (Ref.)	N. Patients	Country	Study Design	Enrollment Period	Males (*n*, %)	Mean Age (SD)	Site of infection (*n*, %)	MDROs (*n*/N, %)	MRSA (*n*, %)	*E. faecium* (*n*, %)	ESBL-Producing Enterobacteriaceae (*n*, %)	CRE (*n*, %)	MDR Nonfermenting Gram- Negative (*n*, %)
Fernandez, 2012 [20]	343	Spain	Prospective cohort	2005–2007; * 2010–2011 **	147 (63) *	60 (13) *	SBP: 159 (46.3); UTI: 139 (40.5) SSTI: 86 (25.1) Pneumonia: 69 (20.1) PB: 45 (13.1) Others 111 (32.4)	98/316 (31) * 40/140 (28.6) **	14 (35) * 6 (35.3) **	14 (31.1) * 11 (39.3) **	46 (32.4) * 12 (21.8) **	0 (0)	23 (92) *
Bartoletti, 2014 [21]	162	Italy	Retrospective cohort	2008–2012	104 (64)	62 (11)	SBP: 13 (8) UTI: 11 (6.8) Pneumonia: 16 (9) PB: 116 (71.6) Others: 6 (3.7)	57/166 (34.3)	6 (28.6)	12 (44.4)	24 (31.2)	14 (18.2)	NR
Bartoletti, 2018 [8]	312	Italy, Spain, Israel, Croatia, Germany	Prospective cohort	2014–2015	204 (65)	61 (12)	SBP: 50 (16) UTI: 35 (11) Pneumonia: 19 (6) PB: 99 (32) Intra-abdominal: 49 (15.7) Others: 60 (19.2)	26/310 (26.1)	12 (27.9)	22 (53.7)	38 (27.9)	9 (6.6)	NR
Fernandez, 2019 [6]	739	13 European Countries	Prospective cohort	2011; 2017–2018	NR	NR	SBP: 130 (25) * UTI: 111 (21.4) * Pneumonia: 85 (16.4) * PB: 28 (5.4) * SSTI: 44 (8.5) * Intra-abdominal: 21 (4.0) * Others: 101 (19.4) *	176/483 (36.4) *	12/30 (40) *	15/44 (34.1) *	36/135 (26.7) *	2/135 (1.5) *	12/13 (92) *
Piano, 2019 [7]	1302	46 centers in Asia, Europe, North and South America	Prospective cohort	2015–2016	898 (69)	57 (13)	SBP: 354 (27) UTI: 289 (23) Pneumonia: 242 (19) PB: 100 (8) SSTI: 101 (8) Others: 216 (17)	322/921 (35.0)	14 (24)	53 (58.2)	131 (35)	35 (9)	NR

* first series; ** second series; MDROs: multi-drug resistant organisms; MRSA: methicillin-resistant *S. aureus*; ESBL: extended-spectrum β-lactamases; CRE: carbapenem-resistant Enterobacteriaceae; SBP: spontaneous bacterial peritonitis; UTI: urinary tract infection; SSTI: skin and soft tissue infection; PB: primary bacteremia; NR: not reported.

**Table 2:** Possible schemes of empirical antimicrobial therapy.

	Community-Acquired Infections	Nosocomial Infections
**Spontaneous bacterial peritonitis**	Ceftriaxone or cefotaxime or amoxicillin–clavulanate or ampicillin–sulbactam	Piperacillin–tazobactam or carbapenem * + anti-MRSA agent **
**Urinary tract infections**	**Uncomplicated**: Fosfomycin or trimethoprim–sulfamethoxazole **Complicated**: Amoxicillin–clavulanate or ampicillin–sulbactam	**Uncomplicated**: Fosfomycin or nitrofurantoin **Complicated**: Piperacillin–tazobactam or carbapenem
**Pneumonia**	Amoxicillin–clavulanate + clarithromycin or azithromycin	Antipseudomonal β-lactam ° + (a fluoroquinolone or an aminoglycoside or colistin) + vancomycin or linezolid (see Refs. [45,46])
**Skin and soft tissue infections**	**Non-necrotizing infections** Amoxicillin–clavulanate + (doxycycline or trimethoprim–sulfamethoxazole or clindamycin) **Necrotizing fasciitis**: Piperacillin–tazobactam or carbapenem + linezolid or (vancomycin or daptomycin + clindamycin)	Piperacillin–tazobactam or carbapenem * + linezolid or (vancomycin or daptomycin + clindamycin)

* A carbapenem (meropenem or imipenem) may be preferred in severe infections in settings with a high rate of ESBL-producing Enterobacteriaceae; ** vancomycin, daptomycin, or linezolid in severe infections; the choice should be based on clinical characteristics of patients (e.g., renal function) and local prevalence of VRE; ° ceftazidime, cefepime, piperacillin–tazobactam, imipenem, or meropenem. See text for details.
